# Comparing ultrasonographically assessed carotid and abdominal aorta plaques in cardiovascular disease risk estimation

**DOI:** 10.1186/s12872-023-03264-1

**Published:** 2023-05-09

**Authors:** Karri Parkkila, Y. Antero Kesäniemi, Olavi Ukkola

**Affiliations:** Medical Research Center Oulu, Research Unit of Internal Medicine, Oulu University Hospital, University of Oulu, Oulu, Finland

**Keywords:** Carotid artery, Abdominal aorta, Plaques, Ultrasound, Cardiovascular diseases

## Abstract

**Background:**

Individual risk estimation is an essential part of cardiovascular (CV) disease prevention. Several imaging parameters have been studied for this purpose. Based on mounting evidence, international guidelines recommend the ultrasound assessment of carotid artery plaques to refine individual risk estimation. Previous studies have not compared carotid artery and abdominal aorta plaques in CV risk estimation. Our aim was to explore this matter in a prospective study setting.

**Methods:**

Participants were part of the Oulu Project Elucidating Risk of Atherosclerosis (OPERA) project. All participants (n = 1007, 50% males, aged 51.3 ± 6.0 years) were clinically examined in the beginning of 1990’s and followed until the end 2014 for fatal and non-fatal CV events.

**Results:**

During a median follow-up of 22.5 (17.5–23.2) years, 246 (24%) participants suffered a CV event and 79 (32%) of those CV events were fatal. When compared to those without plaques, both carotid (hazard ratio, HR 2.854 [95% confidence interval, CI, 2.188–3.721, p < 0.001) and abdominal aorta plaques (HR 2.534 [1.503–4.274], p < 0.001) were major risk factors for CV events as an aggregate endpoint. These associations remained even after adjusting the multivariable models with age, sex, systolic blood pressure, smoking, diabetes, LDL cholesterol, and with previous CV events (coronary artery disease and stroke/transient ischemic attack). However, only carotid plaques were significant risk factors for fatal CV events: multivariable adjusted HR 2.563 (1.452–4.524), p = 0.001. Furthermore, reclassification and discrimination parameters were improved only when carotid plaques were added to a baseline risk model. Adding abdominal aorta plaques to the baseline risk model improved C-statistic from 0.718 (0.684–0.751) to 0.721 (0.688–0.754) whereas carotid plaques improved it to 0.743 (0.710–0.776).

**Conclusions:**

Both carotid and abdominal aorta plaques are significant risk factors for CV events, but only carotid plaques provide prognostic information beyond traditional CV risk factors on fatal CV events. If one ultrasound parameter for plaque detection and CV risk estimation had to be chosen, carotid plaques may be preferred over abdominal aorta.

## Introduction

Atherosclerosis is a dynamic process, and it may take a long time for the molecular and structural changes in the arterial wall to manifest as an ischemic event [1, 2]. Given the nature of atherosclerosis as well as the improvement and availability of imaging methods, it makes sense that researchers have been studying the association between these detectable vascular changes and cardiovascular (CV) events for decades.

The first major ultrasound parameter heavily studied for CV risk estimation purposes was carotid intima-media thickness (IMT), which was first reported to pose an increased CV risk in the Kuopio Ischemic Heart Study [3]. Since then, carotid IMT has been declared a rather poor surrogate marker of atherosclerosis [4], which is mainly due to conflicting findings on its performance over traditional CV risk factors [5, 6] and the several methodological issues related to it [7]. In addition to all previous evidence, our previous publication showed that carotid IMT, indeed, did not improve the prediction of CV events over traditional CV risk factors, whereas abdominal aorta plaques did [8].

Recent findings have shown that the prevalence of subclinical atherosclerosis in non-coronary locations, namely carotid arteries and abdominal aorta and its lower branches, is higher than in the actual coronary arteries [9]. Yet, when it comes to CV risk estimation, the European guidelines do not recommend the ultrasound assessment of peripheral vascular locations other than carotid artery plaque burden [10]. Interestingly, a previous publication reported that femoral plaques are more common and more strongly associated with coronary artery disease compared to carotid arteries [11]. A recently published meta-analysis showed that calcifications in the abdominal aorta were associated with almost 2-fold increased risk for CV events [12]. Another prospective study reported similar effect size for both carotid and femoral plaques in predicting CV events over a 10-year follow-up period [13].

Although several studies concerning subclinical atherosclerosis have been conducted, there are no prospective studies comparing ultrasonographically assessed abdominal aorta and carotid artery plaques and their value in individual CV risk estimation. Our objective was to elucidate this matter in a prospective study setting with a follow-up period of over 20 years.

## Methods

This study is a part of the Oulu Project Elucidating Risk of Atherosclerosis (OPERA) project, the details of which have been previously described [1, 14]. As per the original design of the project, the National Social Insurance Institute’s database was searched for 40–59 years old hypertensive subjects, who were entitled for a special refund for antihypertensive medication. Back in the 1990’s, the National Social Insurance Institute’s blood pressure criteria for the special refund were as follows: diastolic blood pressure over 105 mmHg over a few months follow-up period, or 100 mmHg if the patient already had target organ damage caused by hypertension (e.g., heart failure, renal insufficiency, hypertensive retinopathy). If the patient had family history of CV diseases, had diabetes or severe dyslipidemia, the patient was eligible for the special refund even if their diastolic blood pressure was 100–104 mmHg. If the patients had diabetic or other nephropathy, the diastolic blood pressure limit for the special refund was 95 mmHg. Systolic blood pressure criteria for the special refund were 180 mmHg (in males below 50 years of age and females below 40 years of age) or above 200 mmHg (males over 50 years, females over 40 years). 600 (50% males) hypertensive subjects and their age- and sex-matched non-hypertensive control subjects were randomly selected and asked to participate via an invitation letter (a total of 1200 invitations were sent). Finally, 520 males and 525 females agreed to participate, the overall participation rate being 87%. The OPERA project was approved by the Ethical Committee of the Faculty of Medicine, University of Oulu, and was compatible with the Declaration of Helsinki. A written informed consent was obtained from each participant before participation.

### Clinical examination

The subjects (n = 1,045) participated in an extensive clinical examination in the beginning of 1990’s. All examinations were conducted during the same time of the day (08.00am–01.00pm) and all participants had fasted 12 h before the examination. The clinical examination included anthropologic measures such as height, weight, and the body mass index (weight divided by squared height). Blood pressure measurements were conducted in accordance with the American Society of Hypertension in a sitting position from the right arm with an oscillometric device (Dinamap® model 18465X, Criticon Ltd., Ascot, UK) after a 10–15 min rest. Blood pressure was measured three times at 1-minute intervals and the mean of the last two were used in the analyses. Hypertension was defined as systolic blood pressure of ≥ 140 mmHg and/or diastolic blood pressure of ≥ 90 mmHg.

The participants also completed a detailed questionnaire about smoking habits, alcohol consumption, previous medical history, and medications. The presence of previous coronary artery disease event and previous stroke or transient ischemic attack was determined by acquiring information from the participant and confirming the diagnosis from medical records.

### Laboratory analyses

A more detailed description of the laboratory analyses is provided by Rantala et al. [14]. To determine the true number of diabetic participants, everyone except those with previously known insulin-treated diabetes mellitus completed the oral glucose tolerance test. First, a fasting blood sample was drawn, after which the participants were given 75 g of glucose orally and followed for two hours. Glucose concentrations were measured 1 and 2 h after giving the glucose load. The glucose concentrations were measured using the glucose dehydrogenase method (Diagnostica, Merck, Darmstad, Germany). The participants were considered to be diabetic if their fasting plasma glucose was 7.0 mmol/L or more, or if their 2-hour plasma glucose was 11.0 mmol/L or more. Plasma cholesterol and triglyceride concentrations were determined by enzymatic colorimetric methods (Boehringer Diagnostics, Mannheim GmbH, Germany, catalog nos. 236,691 and 701,912), using a Kone Specific, Selective Chemistry Analyzer (Kone Instruments).

### Ultrasound measurements

The ultrasound measurements have been described earlier [15, 16]. A duplex ultrasound system (Toshiba SSA-270 A, Toshiba Corp., Tokyo, Japan) was used in all ultrasound measurements and all the measurements were performed by a single experienced radiologist, who was blinded to the clinical data. Carotid arteries were assessed using a 7.5 MHz scanning frequency in B-mode, pulsed Doppler mode and color mode. The participant was in a supine position during the measurements with their head turned away from the sonographer at a 45° angle. Both carotid arteries were visualized in anterior oblique and lateral planes, both transversally and longitudinally. The carotid arteries were assessed along their whole anatomical course, starting from just above the clavicle and moving upwards along the common carotid artery, past the carotid bifurcation and eventually along both external and internal carotid artery distally as long as possible. The whole scanning procedure was recorded on a Super VHS videocassette recorder (Panasonic AG-7330, Matsushita Electric Industrial Co., LTD, Osaka, Japan). All measurements were performed about one year later from the video image on the ultrasound device monitor using its electronic calipers by two radiologists. A plaque was defined as moderately or highly echo-dense structure encroaching into the vessel lumen with a distinct area, resulting in IMT more than 50% greater compared to the neighboring sites. The number of plaques was recorded from both carotid arteries along the whole visualized part (including common, external, and internal carotid artery).

The ultrasound examinations of abdominal aorta were carried out according to the same principles as carotid arteries. All examinations were performed by one radiologist blinded to the clinical data. The abdominal aorta and common iliac and femoral arteries were visualized both transversally and longitudinally using a scanning frequency of 5 MHz. The whole scanning procedure was recorded on a Super VHS, and the interpretation of the images was performed about four years later from the video image by two radiologists. The number of abdominal aorta plaques was recorded between the level of renal arteries and the inguinal ligament.

### Study endpoints

The main endpoints of this study were CV events (non-fatal + fatal) and CV mortality. After the baseline clinical examination in the beginning of 1990’s, the participants were followed for over 20 years (until the last day of 2014).

CV event included a major coronary heart disease (CHD) event and stroke (excluding subarachnoid hemorrhage [SAH], whichever of them occurred first. The evidence of CHD was based on the following diagnoses: I20.0, I21, I22 (International Classification of diseases [ICD-10] and Related Health Problems/410, 4110 (ICD-8/9) as the main diagnosis (symptom or cause) and I21, I22 (ICD-19/410 (ICD-8/9) as a first side diagnosis (symptom or cause) or second side diagnosis (symptom or cause) and third side diagnosis (ICD-8/9 only) or if a subject had experienced a coronary artery bypass graft surgery or angioplasty. CHD as a cause of death included I20-I25, I46, R96, R98 (ICD-10/410–414, 798 (not 7980 A) (ICD-8/9) as underlying cause for death or immediate causes of death and I21 or I22 (ICD-10)/410 (ICD-8/9) as first to third contributing cause of death. Stroke (excluding SAH) included I61, I63 (not I636), I64 (ICD-10)/431, 4330 A, 4331 A, 4339 A, 4340 A, 4341 A, 4349 A, 436 (ICD-9)/431 (except 43,101, 43,191), 433, 434, 436 (ICD-8) as main diagnosis (symptom or cause) or as first or second side diagnosis (symptom or cause) or as third side diagnosis (ICD-8/9 only) or as an underlying cause of death or immediate cause of death or as first to third contributing cause of death.

Information on causes of death and events leading to hospitalization was obtained from the Finnish Causes-of-Death Register and the Hospital Discharge Register.

### Statistical analyses

Differences in baseline characteristics between males and females were compared using independent samples T-test (normally distributed variables), Mann-Whitney U-test (skewed variables), and Chi-square test (categorical variables). The proportional hazards assumption for all variables included in the survival analyses was tested using two methods. For categorical variables, Kaplan-Meier curves were created and if the curves overlapped or crossed each other, the proportionality hazard assumption would not have been met. For continuous variables, their partial residuals were obtained using cox regression analysis, and scatter plot images were created of those partial residuals as a function of follow-up time. If there was any non-random pattern in the scatter plot images, the proportionality hazard assumption would not have been met. All variables included in the final analyses met the proportionality hazard assumption.

For the survival analyses, carotid and abdominal aorta plaques were coded into two different variables (four variables in total): (1) carotid plaques, yes or no, (2) carotid plaques, visually binned into three categories (0 plaques, 1 plaque, 2 or more plaques), (3) abdominal aorta plaques, yes or no, and (4) abdominal aorta plaques, visually binned into three categories (≤ 3 plaques, 4–10 plaques, 11 or more plaques). First, the association between the aforementioned variables and CV events and CV mortality was analyzed using the Kaplan-Meier log rank survival functions. Then, carotid and abdominal aorta plaques and their association with the study endpoints were analyzed both individually and together in Cox regression analyses. After these univariate analyses, the Cox regression analyses were adjusted for possible confounding, traditional CV risk factors (age, sex, systolic blood pressure, smoking habits, diabetes, and low-density lipoprotein [LDL] cholesterol). Additional analyses were performed by further adjusting the multivariable model with previous coronary artery disease (CAD) and stroke or transient ischemic attack (TIA) event.

To see whether carotid or abdominal plaques could improve C-statistic, net reclassification index (NRI), and integrated discrimination index (IDI), a baseline risk model was created. The baseline model included age, sex, systolic blood pressure, smoking habits, diabetes, and LDL cholesterol. Then, carotid plaques and abdominal aorta plaques were added to the baseline model and reclassification analyses were run using R Statistics (R Core Team [2021]. R: A language and environment for statistical computing. R Foundation for Statistical Computing, Vienna, Austria. URL https://www.R-project.org/). Both continuous and categorical NRI were used. A 10% cut-off for categorical NRI was used, based on the European guidelines classification of very high CV risk [10].

Other than reclassification analyses, all statistical analyses were performed by using SPSS (IBM Corp. Released 2021. IBM SPSS Statistics for Macintosh, Version 28.0. Armonk, NY: IBM Corp). Statistical significance was set at p < 0.05 (two-tailed).

## Results

The baseline characteristics (Table 1) of our study population are identical to those reported in our previously published work [8]. Overall, 1045 subjects participated in the OPERA study and after excluding 38 subjects due to poor visualization of the abdominal aorta, we were left with a total of 1007 (505 males, 502 females) participants. The mean age of those included in the analyses was 51.3 years, 682 (68%) were hypertensive, 82 (8.1%) had CAD, and 22 (2.2%) had history of stroke or TIA. In general, males had worse CV profile compared to females since males had higher systolic and diastolic blood pressure, they smoked more, they had lower high-density lipoprotein (HDL) cholesterol, as well as higher LDL cholesterol and triglycerides, p < 0.001 for all. A larger proportion of males also had carotid plaques (56% vs. 35%, p < 0.001), but females had more abdominal aorta plaques (85% vs. 90%, p = 0.001). The overall prevalence of subclinical atherosclerotic ultrasound findings in the carotid arteries and abdominal aorta was very high. 915 (91%) of the participants had either carotid or abdominal aorta plaques or both, and only 92 (9.1%) did not have any plaques in neither vascular site. Abdominal aorta plaques were far more common among the participants compared to carotid plaques: 884 (88%) of the participants had visible abdominal aorta plaques, whereas 462 (46%) had visible plaques in either or both carotid arteries. Only 31 (3.1%) of the study subjects had plaques present solely in the carotid arteries and not in the abdominal aorta. Conversely, 453 (45%) individuals who did not have detectable carotid plaques still had one or more plaques in the abdominal aorta.

**Table 1:** Characteristics of the study population

Variable	Males	Females	Total
Number of patients	505	502	1007
Age (years)^†^	50.7 ± 6.0	51.9 ± 5.9	51.3 ± 6.0
BMI (kg/m^2^)^*^	28.0 ± 4.2	27.3 ± 4.9	27.6 ± 4.6
SBP (mmHg)^‡^	152 ± 21.5	144 ± 22.2	148 ± 22.2
DBP (mmHg)^‡^	92.4 ± 11.1	85.6 ± 12.2	89.0 ± 12.2
Hypertension^‡^	386 (76%)	296 (59%)	682 (68%)
Smoking (pack-years)^‡^	11.0 (0.0–25.0)	0.0 (0.0–5.0)	1.0 (0.0–16.0)
Smoking habits^‡^			
Never	158 (31%)	317 (63%)	475 (47%)
Occasionally	5 (1.0%)	7 (1.4%)	12 (1.2%)
Not anymore	186 (37%)	55 (11%)	241 (24%)
Current smoker	156 (31%)	123 (25%)	279 (28%)
Alcohol (g/week)^‡^	63.0 (12.0-144.0)	11.5 (1.0–33.0)	24.0 (2.0–84.0)
Total cholesterol (mmol/l)^*^	5.77 ± 1.06	5.62 ± 1.05	5.69 ± 1.05
HDL (mmol/l)^‡^	1.20 ± 0.31	1.50 ± 0.38	1.35 ± 0.38
LDL (mmol/l)^‡^	3.66 ± 0.95	3.40 ± 0.93	3.53 ± 0.95
Triglycerides (mmol/l)^‡^	1.48 (1.08–2.11)	1.17 (0.91–1.59)	1.31 (0.98–1.84)
Carotid plaques^‡^	285 (56%)	177 (35%)	462 (46%)
Abdominal aorta plaques^†^	430 (85%)	454 (90%)	884 (88%)
Both plaques	260 (52%)	171 (34%)	431 (43%)
No plaques	50 (9.9%)	42 (8.4%)	92 (9.1%)
Diabetes	57 (11%)	43 (8.6%)	100 (9.9%)
CAD	41 (8.1%)	41 (8.2%)	82 (8.1%)
Stroke or TIA	11 (2.2%)	11 (2.2%)	22 (2.2%)
Medication			
ASA^*^	34 (6.7%)	21 (4.2%)	55 (5.5%)
Antihypertensive	256 (51%)	262 (52%)	518 (51%)
Lipid-lowering	19 (3.8%)	9 (1.8%)	28 (2.8%)

The values are means ± SD, medians (1st − 3rd tertile) or number of patients (% within group). The differences in baseline characteristics between males and females were analyzed using independent sample t-test (normally distributed variables), Mann-Whitney U-test (skewed variables) and Chi-square test (categorical variables). BMI, body mass index; SBP, systolic blood pressure; DBP, diastolic blood pressure; HDL high-density lipoprotein; LDL, low-density lipoprotein; CAD, coronary artery disease; TIA, transient ischemic attack; ASA, acetylsalicylic acid. *p < 0.05, †p < 0.01, ‡p < 0.001

**Fig. 1:**
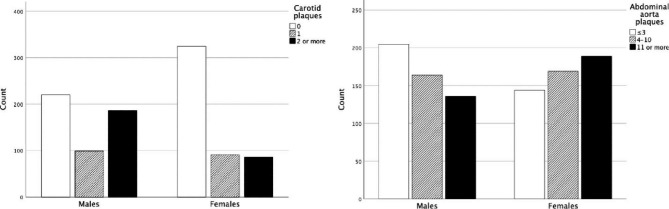
The prevalence of carotid and abdominal aorta plaques in the study population

**Fig. 2:**
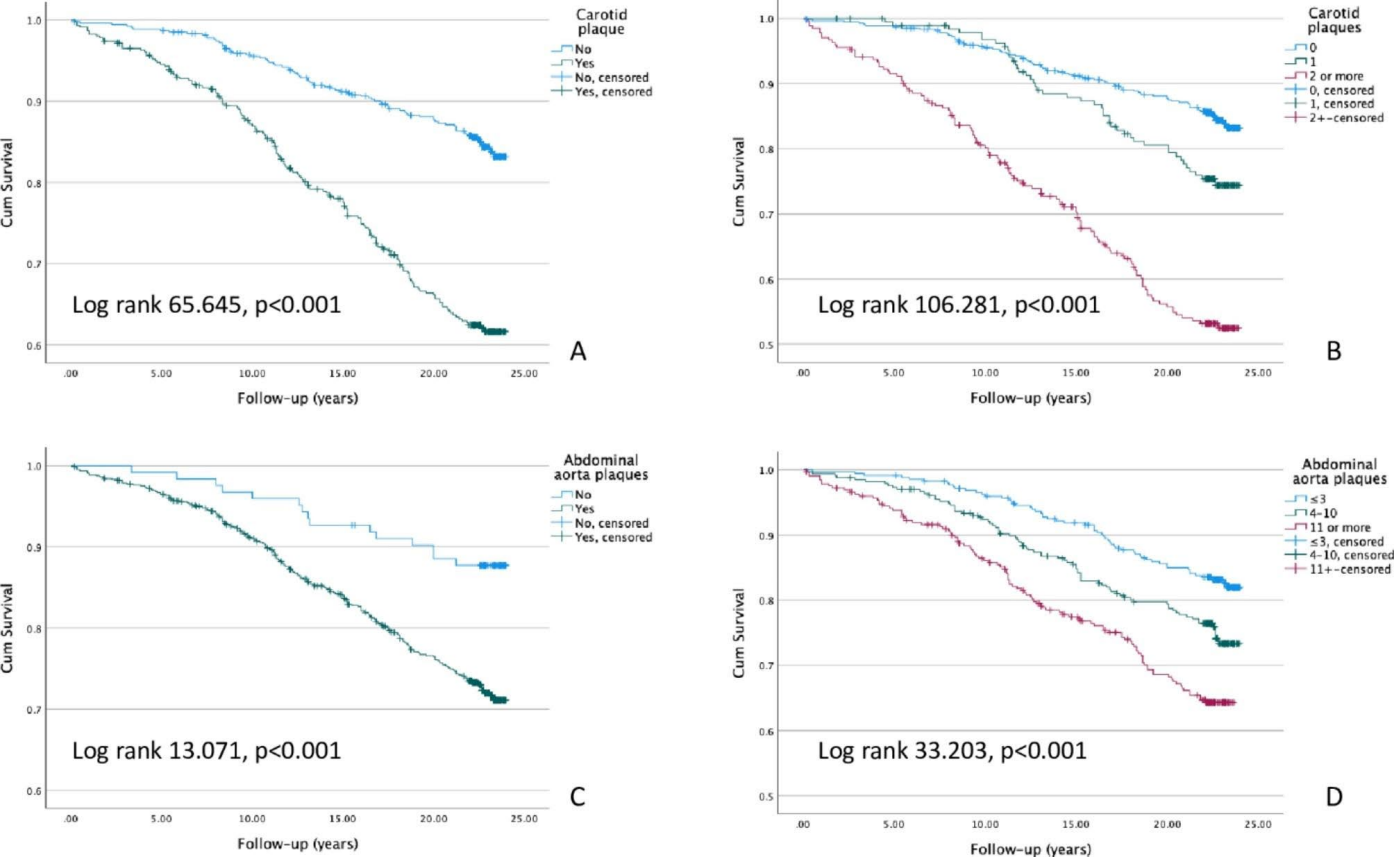
Kaplan-Meier survival curves for cardiovascular events. Carotid plaques present or not (A), carotid plaque tertiles (B), abdominal aorta plaques present or not (C), abdominal aorta plaque tertiles (D). Note the cropped y-axis

**Fig. 3:**
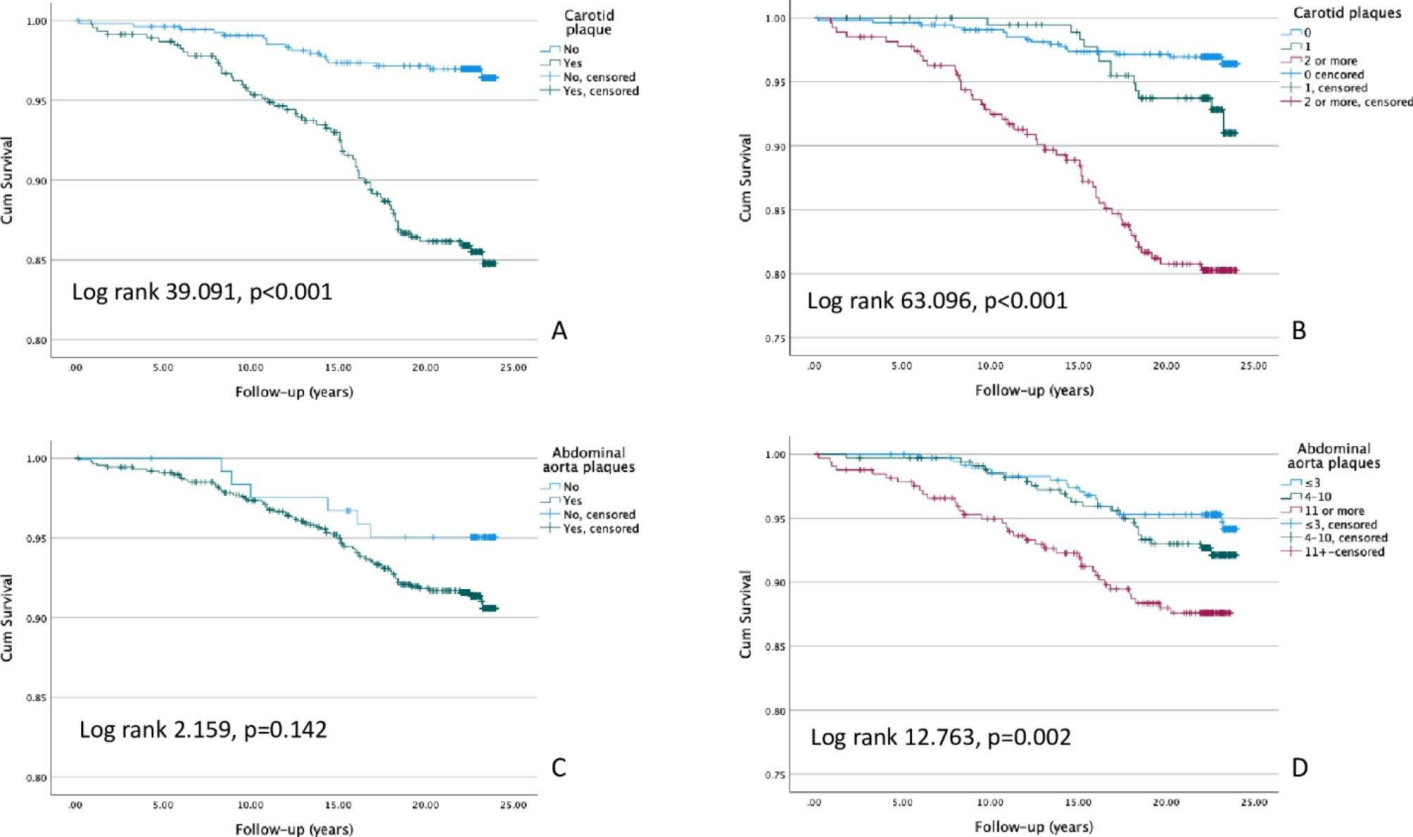
Kaplan-Meier survival curves for cardiovascular deaths. Carotid plaques present or not (A), carotid plaque tertiles (B), abdominal aorta plaques present or not (C), abdominal aorta plaque tertiles (D). Note the cropped y-axis

The median (1st -3rd quartile) follow-up time was 22.5 (17.5–23.2) years, during which 246 (24%) participants suffered a CV event. 79 (32%) of those CV events were fatal. Figure 1 presents the prevalence of carotid and abdominal aorta plaques in males and females separately. Compared to females, significantly larger proportion of males had a (fatal or non-fatal) CV event (33.3% vs. 15.5%) and a fatal CV event (11.5% vs. 4.2%), p < 0.001 for both. When the study population was further categorized in those with and without carotid or abdominal plaques, it was impossible to calculate hazard ratios for both genders independently due to lack of events in females without abdominal plaques (0 CV deaths and 1 non-fatal CV event). Hence, the hazard ratios reported in Table 2 are for both genders together. Unsurprisingly, both carotid and abdominal aorta plaques were associated with a significantly increased risk for adverse CV events during the follow-up period (Figs. 2 and 3). In the adjusted multivariate model, when comparing those who had plaques to those who did not, the increased risk for a CV event (fatal or non-fatal) associated with carotid and abdominal aorta plaques was similar (hazard ratio 1.858, p < 0.001 vs. 1.872, p = 0.023). However, if the study population was divided into tertiles based on their carotid and abdominal aorta plaque counts, and then compared the highest tertiles to the lowest, carotid plaques seemed to be associated with greater CV event risk (2.406, p < 0.001 vs. 1.734, p = 0.008). The difference between the two sites of ultrasonographically detected plaques was even more pronounced when fatal CV events were used as an endpoint in survival analyses. In fact, after adjusting the survival model with age, sex, systolic blood pressure, smoking, LDL cholesterol and diabetes, abdominal aorta plaques lost their significance as a risk factor for fatal CV events (Table 2). On the contrary, carotid plaques posed over 2.7–3.6-fold risk in the adjusted model for a fatal CV event, depending on whether analyzing plaques present vs. absent, or the highest vs. the lowest tertile (Table 2). The effect sizes were similar if the multivariable model was additionally adjusted for earlier CAD and stroke/TIA. Importantly, having no ultrasonographically detectable plaques in carotid arteries or abdominal aorta indicated nearly a halved risk for a composite CV event (no plaques in carotid arteries: HR 0.525 [0.395–0.698], p < 0.001, no plaques in abdominal aorta: HR 0.533 [0.310–0.916], p = 0.023).

**Table 2:** Hazard ratios of carotid and abdominal aorta plaques for adverse cardiovascular outcomes

	Multivariable model 1		Multivariable model 2	
CV events (fatal + non-fatal)	HR (95% CI)	p-value	HR (95% CI)	p-value
*Carotid plaques (yes vs. no)*				
unadjusted	2.854 (2.188–3.721)	<0.001		
adjusted	1.858 (1.397–2.470)	<0.001	1.731 (1.298–2.309)	<0.001
+ abdominal aorta plaques	1.821 (1.370–2.419)	<0.001	1.699 (1.275–2.265)	<0.001
*Abdominal aorta plaques (yes vs. no)*				
unadjusted	2.534 (1.503–4.274)	<0.001		
adjusted	1.872 (1.088–3.222)	0.023	1.879 (1.092–3.232)	0.023
+ carotid plaques	1.764 (1.024–3.036)	0.041	1.795 (1.043–3.089)	0.035
*Carotid plaques (3* ^*rd*^ *vs. 1* ^*st*^ *tertile)*				
unadjusted	3.918 (2.954–5.195)	<0.001		
adjusted	2.406 (1.761–3.287)	<0.001	2.143 (1.558–2.947)	<0.001
+ abdominal aorta plaques	2.317 (1.693–3.170)	<0.001	2.070 (1.503–2.852)	<0.001
*Abdominal aorta plaques (3* ^*rd*^ *vs. 1* ^*st*^ *tertile)*				
unadjusted	2.465 (1.792–3.392)	<0.001		
adjusted	1.734 (1.158–2.597)	0.008	1.605 (1.059–2.431)	0.026
+ carotid plaques	1.601 (1.068–2.398)	0.023	1.476 (0.973–2.239)	0.067
	**Multivariable model 1**		**Multivariable model 2**	
**Fatal CV events**	**HR (95% CI)**	**p-value**	**HR (95% CI)**	**p-value**
*Carotid plaques (yes vs. no)*				
unadjusted	4.721 (2.760–8.075)	<0.001		
adjusted*	2.735 (1.555–4.812)	<0.001	2.563 (1.452–4.524)	0.001
+ abdominal aorta plaques	2.725 (1.549–4.795)	<0.001	2.555 (1.448–4.509)	0.001
*Abdominal plaques (yes vs. no)*				
unadjusted	1.849 (0.804–4.253)	0.148		
adjusted*	1.222 (0.510–2.926)	0.653	1.249 (0.521–2.995)	0.618
+ carotid plaques	1.153 (0.482–2.758)	0.750	1.202 (0.502–2.879)	0.679
*Carotid plaques (3* ^*rd*^ *vs. 1* ^*st*^ *tertile)*				
unadjusted	6.670 (3.840–11.585)	<0.001		
adjusted*	3.639 (1.998–6.628)	<0.001	3.365 (1.835–6.171)	<0.001
+ abdominal aorta plaques	3.485 (1.907–6.367)	<0.001	3.251 (1.766–5.986)	<0.001
*Abdominal aorta plaques (3* ^*rd*^ *vs. 1* ^*st*^ *tertile)*				
unadjusted	2.601 (1.477–4.579)	<0.001		
adjusted*	1.977 (0.970–4.030)	0.061	1.818 (0.865–3.820)	0.115
+ carotid plaques	1.707 (0.847–3.440)	0.135	1.562 (0.751–3.247)	0.233

Cox proportional hazard analyses. HR, hazard ratio; CI, confidence interval; CV, cardiovascular. Multivariable model 1 adjusted for age, sex, systolic blood pressure, smoking habits, diabetes, and LDL cholesterol. Multivariable model 2 adjusted for same variables as in Multivariable 1 + coronary artery disease + stroke/transient ischemic attack

In the reclassification analyses (Table 3), both carotid and abdominal aorta plaques slightly improved the C-index of the baseline model in predicting CV events (fatal + non-fatal). In addition, they both improved the IDI and continuous NRI. As in the survival analyses, carotid plaques had a bigger effect on the reclassification parameters compared to abdominal aorta plaques. However, even though both carotid and abdominal aorta plaques markedly improved continuous NRI in predicting fatal and non-fatal CV events, neither plaque parameter improved the categorical NRI with the cutoff set at 10% (Table 3).

**Table 3:** Reclassification analyses

	C-index (95% CI)	IDI (95% CI)	NRI_cont,_ (95% CI)	NRI_cat_. (95% CI)
CV events (fatal + non-fatal)				
Baseline model	0.718 (0.684–0.751)			
+ carotid plaque burden*	0.743 (0.710–0.776)	0.028 (0.017–0.040) p<0.001	0.415 (0.274–0.556) p<0.001	0.017 (-0.007–0.040) p=0.168
+ abdominal aorta plaque burden†	0.721 (0.688–0.754)	0.004 (-0.001–0.009) p=0.104	0.171 (0.029–0.314) p=0.019	0.008 (-0.007–0.023) p=0.303
+ carotid plaques (yes or no)	0.734 (0.700–0.767)	0.016 (0.008–0.024) p<0.001	0.526 (0.388–0.663) p<0.001	0.002 (-0.027–0.031) p=0.898
+ abdominal aorta plaques (yes or no)	0.722 (0.689–0.756)	0.004 (0.000–0.008) p=0.047	0.05 (-0.077–0.177) p=0.441	0.003 (-0.015=0.020) p=0.775
**Fatal CV events**				
Baseline model	0.767 (0.714–0.820)			
+ carotid plaque burden*	0.801 (0.754–0.848)	0.035 (0.011–0.038) p<0.001	0.702 (0.486–0.917) p<0.001	0.089 (-0.021–0.198) p=0.112
+ abdominal aorta plaque burden†	0.768 (0.716–0.821)	0.004 (-0.002–0.010) p=0.195	0.221 (-0.007–0.449) p=0.057	-0.018 (-0.063–0.027) p=0.434
+ carotid plaques (yes or no)	0.791 (0.743–0.839)	0.015 (0.005–0.025) p=0.003	0.608 (0.404–0.813) p<0.001	0.098 (-0.008–0.204) p=0.069
+ abdominal aorta plaques (yes or no)	0.768 (0.715–0.821)	0.000 (-0.002–0.019) p=0.897	0.022 (-0.178–0.222) p=0.832	-0.003 (-0.010–0.003) p=0.317

Baseline model: age, sex, systolic blood pressure, smoking, LDL cholesterol and diabetes. CI, confidence interval; IDI, integrated discrimination index; NRIcont., net reclassification index (continuous); NRIcat., net reclassification index (categorical, cutoff 10%). *carotid plaque burden was visually binned into three groups of equal percentiles based on scanned cases: 0 plaques, 1 plaque, and 2 or more plaques. †femoral plaque burden was visually binned into three groups of equal percentiles based on scanned cases: 0–3 plaques, 4–10 plaques, and 11 or more plaques

## Discussion

First, the results in this paper add on to the accumulating evidence of the considerably high prevalence of subclinical atherosclerosis in a middle-aged population. Second, the results of this study are demonstrating that both carotid and abdominal aorta plaques are, unsurprisingly, associated with adverse CV events in a middle-aged population over a two-decade-long follow-up period. Both vascular sites seemed to improve the prediction of a composite endpoint of fatal and non-fatal CV outcomes over traditional CV risk factors. On the contrary to carotid plaques, however, abdominal aorta plaques lost their significance as a predictor of fatal CV events after adjusting for common CV risk factors.

The Progression of Early Subclinical Atherosclerosis (PESA) is a landmark study showing the high prevalence and vascular distribution of subclinical atherosclerosis in apparently healthy middle-aged population. The PESA study included over 4000 subjects without CV or other conditions affecting life-expectancy, and the results showed that 63% of the subjects had atherosclerotic plaques either in coronary, carotid or ilio-femoral arteries or in abdominal aorta [9]. Importantly, they showed that subclinical atherosclerosis is far more common in arteries other than coronary: 5% of females and 25% of males had coronary calcification, whereas 29% of females and 53% of males had plaques in ilio-femoral arteries. Subclinical atherosclerosis was even more prevalent in our study population: 35% of females and 56% of males had plaques in carotid arteries whereas a whopping 90% of females and 85% of males had plaques present in abdominal aorta. Our study population was not free of CV conditions at baseline (e.g., 68% had hypertension), which obviously is the most important factor explaining the differences seen in the prevalence of subclinical vascular changes between our studies.

Several studies have shown the predictive value and additional information that ultrasound assessment of carotid artery plaques may entail as it pertains to CV risk estimation [17–19]. A multi-ethnic study that included nearly 3000 low-risk participants (coronary artery calcium score 0) showed that the presence of ultrasonographically detected carotid plaques was associated with an increased risk of later incidence of coronary artery calcium as well as coronary heart disease events [17]. Interestingly, the authors reported that the presence of carotid plaques was not associated with stroke or total CV event risk. Moreover, the authors did not detect improvements in C-statistic, discrimination, or reclassification parameters if carotid plaques were added to a risk model containing traditional CV risk factors [17]. On the contrary, our results indicate that carotid plaques carry prognostic information beyond traditional risk factors on both fatal and non-fatal CV events. In addition, our results showed that by adding carotid plaques to a risk model consisting of traditional CV risk factors (age, sex, systolic blood pressure, smoking, diabetes, LDL cholesterol), the C-statistic increased from 0.718 (0.684–0.751) to 0.743 (0.710–0.776). Carotid plaques also improved reclassification (continuous NRI 0.4149 [0.2738–0.5560], p < 0.001) and discrimination (IDI 0.028 [0.017–0.040], p < 0.001). The major difference between our studies is that Mehta et al. included a highly selective group of participants screened to be a “low-risk” population, whereas our study population consisted of individuals with a more high-risk CV profile (68% were hypertensive, 8.1% had suffered a coronary event etc.). Together these findings would suggest that, on one hand, carotid plaques might be a highly informative clinical parameter indicating future risk of CV events in an unselected population not entirely free of CV conditions. On the other hand, carotid plaque screening of individuals with a (reliably detected) low CV event risk seems, from a clinical standpoint, redundant.

Albeit studied to a lesser extent, vascular sites other than carotid arteries have been shown to be potential CV risk modifiers. For example, plaques in femoral arteries have been reported to associate with future risk of CV events equal to carotid arteries [13, 20–22]. Independent meta-analyses have also concluded that subclinical atherosclerotic changes in abdominal aorta are strongly associated with CV events [12, 23]. In our previous publication [8], we demonstrated that abdominal aorta plaques were more accurate predictors of future CV risk compared to carotid IMT. We further speculated that this finding may be simply explained by the fact that intimal thickening represents the earliest stage of the atherosclerotic process, whereas a visible plaque is a manifestation of a more advanced atherosclerotic lesion. Therefore, detecting plaques in abdominal aorta would indicate a more advanced phase of the atherosclerotic vascular disease compared to carotid intima-media thickening, which is probably why aortic plaques were statistically stronger CV risk indicators compared to carotid IMT [8]. No previous study has, however, compared the predictive value of carotid and abdominal aorta plaques in CV risk estimation. Based on our results, plaques in carotid and abdominal aorta pose a similar risk for CV events as an aggregate endpoint (non-fatal + fatal), but only carotid plaques are associated with fatal CV events in multivariable analyses. Adding carotid plaques to the risk model consisting of traditional CV risk factors also provided much greater improvements in both reclassification and discrimination analyses compared to abdominal aorta plaques, thus making carotid plaques a superior indicator of underlying CV disease risk. Although highly speculative, our results might be explained simply by the smaller caliber of carotid artery and its physiological role in supplying brain blood flow, thus making it more vulnerable to obstructing processes which may lead to potentially fatal cerebrovascular events. To hypothesize further, the different embryonic origins of carotid arteries and abdominal aorta may play a role in their susceptibility to atherosclerosis and in the clinical manifestations related to plaque buildup.

Atherosclerosis is known to be a systemic disease and there exists an increased risk of CV events along with the increasing number of atherosclerosis-affected vascular locations [24, 25]. Ultrasound imaging of the vasculature may also help in detecting high-risk patients (i.e., patients needing more intense preventive measures) that would have otherwise been missed if the risk assessment is based only on validated risk scores [26]. With these findings in mind, it is safe to say an extensive ultrasound examination of a patient in a CV disease prevention setting should not be overlooked. If one location had to be chosen for some reason, our results suggest that carotid arteries should be preferred over abdominal aorta. Of note, neither carotid nor abdominal aorta plaques improved patient reclassification over the 10% risk threshold which classifies apparently healthy people into very high CV disease risk group, according to European guidelines [10]. This means that detecting carotid or abdominal aorta plaques might not significantly affect clinical decision-making regarding CV disease prevention. Therefore, CV risk estimation should, obviously, not be driven by vascular ultrasound findings alone, but, instead, should be a synthesis of clinical findings, patient preferences, and the expertise of the physician.

Some limitations are related to our study. First, our study population is relatively small compared to many studies conducted in recent years. Since our participants were recruited in the 90’s, the hypertension criteria used back then were relatively high, and a larger proportion (68%) of our participants are hypertensive in today’s criteria than was originally intended. Also, our study population was not completely free of previous CV events (8.1% had experienced a CAD event and 2.1% a stroke/TIA event) which hinders the interpretation of our results in a pure primary prevention setting (although the multivariable models were adjusted with previous CV events). When it comes to the parameters used in this study, the number of visible plaques is a very crude and simplistic parameter to be used in CV risk estimation. Although the most simple and reproducible clinical parameters are usually the most useful in a clinical setting, individual plaque qualities (i.e., severity of stenosis, plaque vulnerability) might provide more accurate information about the risk of thrombus formation compared to the crude parameters used in this study [2]. Moreover, the plaque assessments were performed thirty years ago and the ultrasound techniques have greatly improved, especially in assessing the aorta, which could affect the obtained effective value of abdominal aorta plaques in the 1990’s compared to present day. Lastly, our study population consisted of Caucasian, middle-aged, Scandinavian subjects only, which obviously prevents any generalizations of our findings to individuals of other ethnicities.

## Conclusions

This is the first prospective study comparing ultrasonographically assessed carotid and abdominal aorta plaques in CV disease risk estimation in a very long follow-up period spanning over two decades. Both carotid and abdominal aorta plaques are strong risk factors for CV events in a population not entirely free of previous CV events. However, only carotid plaques provided prognostic information beyond traditional CV risk factors on individual risk of fatal CV events. If one ultrasound parameter should be chosen to complement CV risk estimation, carotid plaques may be preferred over abdominal aorta plaques.

## Data Availability

The dataset used and analyzed during the current study are available from the corresponding author on reasonable request.

## Abbreviations

CV: Cardiovascular
IMT: Intima-media thickness
OPERA: Oulu Project Elucidating Risk of Atherosclerosis
SAV: Subarachnoid hemorrhage
ICD: International Classification of Diseases
CAD: Coronary artery disease
TIA: Transient ischemic attack
LDL: Low-density lipoprotein
NRI: Net reclassification index
IDI: Integrated discrimination index
HDL: High-density lipoprotein
PESA: The Progression of Early Subclinical Atherosclerosis
